# The Present Epidemic of Influenza

**Published:** 1890-03

**Authors:** Louis Fisk Bryson

**Affiliations:** New York


### THE PRESENT EPIDEMIC OF INFLUENZA.

BY LOUIS FISK BRYSON, M. D., NEW YORK.

Since the year 1510 there have been distinctly recognizable epidemics of influenza, occurring on an average, once in about ten years. Their duration is from four to six weeks, though they may last nine or ten months. The mortality is two per cent. In view, however, of the immense numbers attacked, this small per cent. is sufficiently appalling in point of actual death rate. The prognosis is good, barring certain conditions that equal numerically the exceptions to a rule in French grammar. One seizure does not secure immunity from a second.

The real cause of influenza is unknown. Any theory is admissable since no two experts agree, and whole communities are at variance in regard to it. There are food diseases, house diseases,—why not air diseases? Or, to speak more exactly, electric diseases? Epidemic influenza may be due to some astronomical influence that upsets temporarily the normal electric equation. Certain physical phenomena quite capable of altering in marked degree atmospheric electricity have been present during and shortly before the epidemic, among them earthquakes and excessive humidity. Earthquakes may produce extraordinary electric phenomena. The modes of motion —heat, light, electricity—are correlated to each other, and to mechanical motion or the motion of matter in the mass. The general accepted theory of earthquakes, at present ascribes the cause to terrestrial gravitation or condensation of the earth, by which the mass moves slowly towards the centre. This motion is partially arrested by the resistance of matter to further condensation, and so molar motion is converted into molecular motion, manifested as heat and electricity. Evaporation being the source and some impurity in water, a necessity of the evolution of air electricity, the conditions favoring an increase of this fluid have greatly prevailed of late. In seasons that do not swerve from their natural course, the wastes of the summer are gathered into the soil in the fall, and there decaying, are carried away and neutralized, if the ground be dry and air can find its way into it. But where water is, air cannot penetrate. Remove the water and air follows, forced by the pressure of fifteen pounds to the square inch, carrying with it the warmth of the sun, purifying, vivifying, and vitalizing the ground. Heat, moisture and impurity are necessary elements in the production of infectious disease, though neither of the three, or the three combined, can create the morbid fitness needed for its appearance and propagation, unless there be present a fourth constituent, which thus becomes the essential factor. This something may yet be found to be an altered condition of atmospheric electricity, capable of causing corresponding derangement of the nervo-electric fluid. There is a departure, in consequence, from the natural equilibrium of atmospheric and terrestrial electricity that brings about a corresponding disturbance of the normal electric balance in the human body.*

So long as any excess of electricity is neutralized by suitable atmospheric conditions, it remains without injury to health. But if the earth is highly excited negatively, the air being also in high negative tension, the natural positive of the human body having been abstracted, and leaving an excess of high tension negative struggling to coalesce with suitable outside fluid, what becomes of poor humanity? It is supposable that the impression of irritant and untoward electricity falls, in our present epidemic, upon the nervous centres and upon the pneumogastric nerve, chiefly upon its pulmonary, gastric and cardiac branches, causing excitation first, then nerve exhaustion or paralysis in greater or less degree, according to the condition of the nerves as to susceptibility, receptivity, or powers of resistance. It is not impossible that this peculiar nervous irritation may produce the phenomena of grippe, by means of some natural ptomaine, and also be the cause of other epidemics that closely resemble it. Some have thought the present epidemic to be a form of dengue. Others suggest that its earlier stages closely resemble cerebro-spinal meningitis. Dengue and epidemic influenza bear such a close relationship that it is reasonable to mistrust they are first cousins. The

* See New Orleans Medical and Surgical Journal, July, 1881.

same sudden loss of strength, of appetite, and of mental force exist in both. There are frontal pains, conjunctivities, and the quick appearance. Except for the invariable presence of intense pain about the joints, the symptoms read like those of grippe, which are the following, existing together or only in part: Brief malaise, headache, chill, fever, pain over the whole body, especially in the back and loins, suffusion of the eyes, bronchitis, a characteristic cough, gastro-intestinal irritations, skin eruptions, delirium, great prostration, physical and mental; and certain violent neuralgias. The pain of dengue is something that is never forgotten, and described as the sum total of human suffering. So, too, the headache in grippe, even by women familiar with the pangs of childbirth. A troublesome cough in the earlier stages of dengue; may remain and annoy long after the fever has subsided. With the exception of epidemic influenza, there are but few diseases expressing themselves in so many ways as dengue, or are subject to such serious complications and sequelæ. The nervous centres suffer much in severe forms, and the mild ones often present cerebral lesions and neurotic complications. Later pulmonary abnormities can be traced to dengue. The disease in Bengal was very often supposed to develop into a form of phthisis. The most striking similarity of dengue and epidemic influenza is the fact that, though the patients are cured, they do not always get well. These epidemics, as remote causes of deaths, offer an interesting field for future research. Stages resembling the three stages of dengue are often present in grippe, viz., two periods of fever separated by two or three days remissions. It may be assumed that many so-called relapses, after apparently light attacks of the present epidemic, are in reality the third stage of influenza, following the period of remission. Mild seizures run their course in from four to seven days—moderate use of diaphoretics and regulated diet—without the development of any second fever. In cases marked by gastric irritation and depression of nerve vitality, the disease lasts from twenty-one to twenty-eight days. The final termination is either by disappearance of the symptoms,—local as well as general—or by some critical crisis, as increased secretion of mucus, urine, or perspiration, or by diarrhœa. When death occurs, it may be due to one or all of the following causes: (a) to paralysis of the lung or to capillary bronchitis, especially in the aged; (b) to pneumonia; (c) to cerebral congestion, to apoplexy caused by violent coughing, or to profound exhaustion of the nerve centres.

Treatment aims to secure three distinct and important results: I, to unlock and stimulate secretion; II, to counteract the evil effects of nervous exhaustion; and, III, to maintain nutrition during convalescence. Calomel, aconite, tartar emetic, ipecac, valerion, nux vomica, digitalis, hyoscyamus, camphor, morphia, and lactucarium are remedeis of well known efficiency that the experience of the past few weeks has confirmed. Alcohol is interdicted, except for emergencies. Cod liver oil, as a food and tonic in late stages, is not well borne. Malt extract, quinine, and cocoa, and beef wine and iron are of far greater value. The Turkish bath, massage, and electricity are standard resources during the frequently long convalescence. Diminished chest expansion requires immediate attention, in the form of systematized mechanical exercise. For this purpose, the respirator used in the establishment of Dr. Henry Ling Taylor is probably the best means known. Failing this, the same passive movements made by a trained attendant, according to the rules laid down by Ling, will be of great value. New impressions in this way may be sent to the brain, and thus is secured an advantageous redistribution of nervous energy. Obstinate cough that defies remedies as ammoniæ muriaticum, sal ammoniæ, hyoscyamus, and preparations of lactucarium, will disappear under the influence of a change of climate.

Grippe being an insidious dastardly thing, at best, whoever has an attack should go at once to bed and stay there five days, regardless of sentiments or sensations. As long as any sense of fatigue or perpetual weariness remains, it is unwise to undertake the real business of life, for the period of convalescense is fraught with unknown, yet certain, danger, that may at any time assume a local habitation and a name. Epidemic influenza has a powerful alterative effect upon the whole organism. It is the frequent starting-point of distressing conditions of which phthisis and insanity are the most formidable.

